# Assessment of Quality of Life and Difficulties in Recording Data from Health-Related Quality of Life Questionnaires in Patients with Cancer Undergoing Immunotherapy Treatment

**DOI:** 10.3390/healthcare13091002

**Published:** 2025-04-26

**Authors:** Laura Bibiano Guillén, Cristina Recio Carrasco, José Miguel Cárdenas Rebollo, Dihan van Niekerk, Jesús Rodríguez Pascual, María Carmen Rubio-Rodríguez, Miguel A. Reina

**Affiliations:** 1 Department of Oncology, HM Puerta del Sur Hospital, 28938 Madrid, Spain; criseltrebol@gmail.com; 2 Department of Mathematics and Data Science, CEU-San-Pablo University, 28925 Madrid, Spain; cardenas@ceu.es; 3 Department of Physiological Science, University of Stellenbosch, Stellenbosch 7602, South Africa; vanniekerkdihan@gmail.com; 4 Department of Medical Oncology, Vithas Hospital Group, 28043 Madrid, Spain; rodriguezpje@vithas.es; 5 Department of Radiation Oncology, HM Hospitales, 28050 Madrid, Spain; crubio@hmhospitales.com; 6 Department of Anesthesiology, School of Medicine, CEU-San-Pablo University, 28925 Madrid, Spain; miguelangel@perticone.e.telefonica.net; 7 Department of Anesthesiology, College of Medicine, University of Florida, Gainesville, FL 32610, USA

**Keywords:** immunotherapy, oncology, quality of life, fatigue, nausea, vomiting, pain, dyspnea

## Abstract

**Background:** Prospective studies evaluating the challenges of systematically assessing health-related quality of life in patients with cancer outside clinical trials are lacking. This study aimed to evaluate the quality of life of patients with cancer treated with immunotherapy such as checkpoint inhibitors and to determine the difficulties and limitations in achieving data collection from health-related quality of life questionnaires. **Methods:** We carried out a prospective observational study over 15 months in 30 patients with solid tumors undergoing checkpoint inhibitor therapy in an outpatient setting. We assessed health-related quality of life using the European Organization for Research and Treatment of Cancer QLQ-C30 quality of life questionnaire at treatment initiation, three months, and six months. We analyzed compliance rates, reported difficulties, and treatment-related toxicities. **Results:** Of the 30 patients, 26 completed the health-related quality of life standardized questionnaire at one month (86.6%), 24 at three months (80%), and 18 at six months (56.6%). Patients receiving checkpoint inhibitor monotherapy showed an improvement in global health status scores from 60 at baseline to 65 at three months and 70.8 at six months. These findings suggest that checkpoint inhibitor therapy delays symptom onset and positively impacts quality of life. Fatigue was the most frequently reported adverse effect, followed by pain, dyspnea, and gastrointestinal symptoms. **Conclusions:** Checkpoint inhibitor treatments may delay the onset of cancer-related symptoms, positively influencing patient-reported health-related quality of life (HRQoL) outcomes. However, this study highlights significant methodological challenges in collecting standardized HRQoL questionnaire data outside of clinical trials, including declining patient compliance over time. These findings underscore the need for adapted HRQoL assessment strategies tailored to the unique treatment trajectories of immunotherapy patients.

## 1. Introduction

Oncological treatments that adjust the immune response against tumor cells, collectively known as immunotherapy, have recently transformed cancer treatment strategies [1]. When administered either alone or in combination with chemotherapy (ChT), these therapies have changed the toxicity profile of conventional cancer treatments. A key category within immunotherapy is checkpoint inhibitors (CPIs), drugs designed to block specific immune checkpoint proteins produced by immune cells such as T cells and specific tumor cells [2]. These checkpoint proteins function as negative regulators of the immune response, and tumor cells exploit this mechanism to evade immune detection, thereby promoting disease progression. CPIs counteract this tumor-protective mechanism by restoring immune system activity, leading to tumor cell destruction [3]. Current oncological treatment strategies incorporate immunotherapy alongside surgery, radiotherapy, ChT, and targeted therapies [2], positioning immunotherapy as the fifth pillar of cancer treatment [4]. Despite the clinical benefits of CPIs, their mechanism of action can lead to immune-related adverse effects. While the incidence of these toxicities can be considerable, their overall toxicity profile is generally more favorable than that of standard ChT, often resulting in an improved quality of life for patients [5,6,7]. Assessing patients with cancer quality of life (QoL) is essential for optimizing treatment strategies tailored to individual patient conditions and disease progression. A comprehensive approach also necessitates enhanced communication between physicians, nurses, and patients. Patients with cancer must contend not only with their disease but also with the side effects of treatment; thus, understanding their QoL is critical for the personalization of therapy. Although clinical trials often include assessments of health-related quality of life (HRQL) in patients undergoing immunotherapy with or without ChT [8,9], there is limited prospective evaluation of the challenges and constraints associated with these assessments outside the controlled setting of clinical trials. This study aims to identify the challenges and limitations associated with collecting HRQL data through patient-reported questionnaires. Additionally, it seeks to analyze HRQL scores in patients receiving CPIs, either alone or in combination with ChT, while also evaluating the emergence of potential toxicities.

## 2. Materials and Methods

### 2.1. Study Design

A prospective, observational, and descriptive study was conducted on 30 patients undergoing treatment with checkpoint inhibitors (CPIs) between 1 October 2021, and 21 December 2022. Data were collected at three time points: at immunotherapy treatment initiation using CPIs and three and six months post-treatment. The study was conducted at the HM Group Hospitals in Madrid, Spain.

### 2.2. Inclusion and Exclusion Criteria

Patients eligible for inclusion were adults (≥18 years) diagnosed with solid neoplasms, excluding hematological malignancies, who had not previously received immunotherapy.

### 2.3. Questionnaire and Variables

The validated European Organization for Research and Treatment of Cancer QLQ-C30 quality of life questionnaire was used to assess health-related quality of life (HRQL). The variables analyzed are detailed in Table 1. The questionnaire was administered at baseline (before treatment initiation) and at, 3, and 6 months post-treatment.

The QLQ-C30 consists of 30 questions evaluating five functional domains (physical, emotional, role, social, and cognitive status), three symptom scales (fatigue, pain, and nausea and vomiting), a global health status/quality of life scale, and six additional single-item symptoms (dyspnea, insomnia, anorexia, constipation, diarrhea, and financial difficulties).

Responses were recorded using a Likert-type scale. Functional and global health status scales were scored such that higher values indicated a better quality of life, whereas higher scores on the symptom scales corresponded to a more significant symptom burden and reduced quality of life. Scores were standardized on a scale from 0 to 100 to quantify the impact of immunotherapy on HRQL.

An oncology nurse supervised the questionnaires, who explained previously in detail the meaning of each of the questions and patients completed them independently, with assistance from a family member or a nurse if necessary. All difficulties that arose related to filling out the questionnaires were also recorded by the nurses.

Compliance rates were defined as the proportion of patients who completed the questionnaire at each follow-up point over six months. Patients with complete data at the completion of the study were included in the analysis, with documentation of any withdrawals due to disease progression, loss to follow-up, or death.

### 2.4. Data Analysis

Descriptive statistics were used to summarize patient data, including means, medians, absolute frequencies, relative frequencies, and value ranges.

We tested the normality of the groups using the Kolmogorov–Smirnov test with Lilliefors correction and the Shapiro–Wilk test. As the data did not follow a parametric distribution, the Kruskal–Wallis test was used to compare groups. Post hoc analysis was conducted using the Mann–Whitney U test when significant differences were identified. Statistical significance was set at *p* ≤ 0.05.

All statistical analyses were performed using IBM SPSS Statistics for Windows, Version 28.0 (IBM Corp., Armonk, NY, USA).

### 2.5. Supplementary Data Collection

In addition to the questionnaire responses, medical records were reviewed to collect clinical data on incidents that could affect the validity of questionnaire data, including:

- Tumor histology;
- Disease extent;
- Primary tumor location;
- Treatment intent (radical, neoadjuvant, adjuvant, or palliative);
- Type of CPI used;
- Presence or absence of symptoms;
- Baseline assessment of health and quality of life (one week before treatment initiation);
- Hospital admissions;
- Immunotherapy treatment cycle delays due to the patient’s condition.

### 2.6. Ethical Considerations

All participants were informed about the voluntary nature of their participation, their right to withdraw at any time without explanation, and the confidentiality of their responses. Data were de-identified for reporting purposes, and all recorded information was encrypted and securely stored with restricted access.

This study was approved by the Grupo Hospital Madrid Clinical Research Ethics Committee (CEIC code 22.03.1842E2-GHM).

## 3. Results

Of the 30 patients (17 men and 13 women) who initiated treatment, 26 completed the questionnaire at one month, 24 at three months, and 18 at six months, corresponding to compliance rates of 86.6%, 80%, and 56.6%, respectively. Patient discontinuation was attributed to disease progression, requiring a change in treatment and/or patient death. The mean age was 63 years (39–83 years). All patients were prescribed checkpoint inhibitor (CPI) therapy.

Among the cohort, 70% (*n* = 21) had metastatic disease, and 43.3% (*n* = 13) were receiving treatment with palliative intent. The most frequently observed tumor types were lung (46.6%), breast (13.3%), kidney (6.6%), and bladder cancer (6.6%). Additionally, 40% (*n* = 12) of patients had undergone prior chemotherapy (ChT) before study inclusion, while 60% (*n* = 18) had not.

Of the 30 patients, 60% (*n* = 18) received CPI monotherapy, whereas 40% (*n* = 12) received ChT in combination with CPI drugs. Patient characteristics are presented in Table 2. Questionnaire completion rates remained high through the three-month assessment, declining at the six-month follow-up.

The median follow-up duration was 4.8 months. By study completion, 50% of patients (*n* = 15) remained enrolled, while 26.6% (*n* = 8) had died, and 23.3% (*n* = 7) discontinued participation due to disease progression (*n* = 4), poor medication tolerance (*n* = 1), or unknown reasons (*n* = 2).

### 3.1. Challenges in Completing HRQL Questionnaires

Difficulties in completing the questionnaire were identified in 3.3% (*n* = 1) of patients with cognitive impairment, 3.3% (*n* = 1) with an intellectual disability, and 20% (*n* = 6) with reading or writing difficulties.

Regarding hospital admissions, 16.6% (*n* = 5) were hospitalized at baseline, 16.6% (*n* = 4) at three months, and 35.2% (*n* = 6) at six months. Additional challenges included treatment cycle delays due to worsening clinical status or disease progression, affecting 11.5% (*n* = 3) of patients at one month, 20.8% (*n* = 5) at three months, and 52.9% (*n* = 9) at six months.

### 3.2. Health-Related Quality of Life (HRQL) Analysis

Among patients assessed using the QLQ-C30 questionnaire, 53.3% were aged ≥65, and 46.6% were <65. The global health status/quality of life (QoL) scores for these two groups were 60.4 and 68, respectively, with no statistically significant difference (*p* = 0.39), either at three months (*p* = 0.95) or six months (*p* = 0.17).

Patients with prior ChT exposure (40% of the sample) had a lower baseline global health status score (56.2) than those without prior ChT (score: 68.2). However, this difference was not statistically significant (*p* = 0.23) at baseline, three months, or six months. Over time, patients with prior ChT exhibited improvements in QoL scores, increasing to 63.5 at three months and 66.6 at six months, whereas those without prior ChT maintained scores of 64 (*p* = 0.90) at three months and 70.5 (*p* = 0.58) at six months.

At baseline, patients who had received prior ChT exhibited a higher symptom burden, particularly in fatigue, nausea and vomiting, pain, and dyspnea, correlating with lower QoL scores. However, by three and six months (Table 3), these patients showed reduced symptom severity and improved QoL scores. In contrast, patients without prior ChT reported increased symptom burden at three months, though their symptom scores improved at six months, suggesting a delayed adaptation period.

### 3.3. Impact of Disease Extent and Treatment Type on QoL

At baseline, disease extent (localized, locally advanced, or metastatic) was associated with a statistically significant difference in QoL scores (*p* = 0.045). However, this difference was insignificant at three months (*p* = 0.99) or six months (*p* = 0.35).

Patients receiving CPI monotherapy achieved a global health status score of 65 at three months (62.5% of the sample), which improved to 70.8 at six months (66.6% of the sample). Those receiving ChT in combination with CPI reported a score of 62 at three months (37.5% of the sample), which improved to 66.6 at six months (33.3% of the sample). While both groups exhibited good QoL outcomes, no statistically significant differences were observed between them at three (*p* = 0.80) or six months (*p* = 0.66).

The most commonly reported symptoms were fatigue, pain, constipation, and dyspnea, which were consistently the highest-scoring symptom scales across both groups (Table 4).

Patients receiving CPI monotherapy demonstrated improvements in fatigue at six months, while both groups showed reductions in dyspnea over time.

As shown in Table 5, patients experienced improvements in QoL following CPI therapy, whether administered alone or in combination with ChT.

## 4. Discussion

This study prospectively evaluated the challenges and limitations associated with recording health-related quality of life (HRQL) data and assessing potential treatment-related toxicities in 30 cancer patients receiving immunotherapy outside the controlled setting of clinical trials. This real-world approach provides a broader perspective on the complexities of oncology care, capturing patient-reported outcomes in routine clinical practice rather than under the rigid conditions of a clinical trial.

Quality of life (QoL) is a subjective measure influenced by physical, social, and psychological well-being, and it is increasingly recognized as a key indicator of treatment effectiveness [8,10]. CPIs have significantly improved survival rates in metastatic tumors [11,12,13]; however, it remains crucial to determine whether these therapies delay the onset of symptoms and result in measurable improvements in QoL in real-world contexts.

Beyond objective clinical outcomes, patient-reported improvements in well-being, symptom relief, and treatment effectiveness must be systematically quantified through standardized HRQL tools, such as the QLQ-C30 questionnaire. These assessments contribute to clinical decision-making, ensuring that subjective patient experiences inform personalized treatment strategies. In this context, oncology nurses play a pivotal role in identifying treatment-related toxicities and monitoring QoL trajectories, thus facilitating optimized care planning.

In this study, comprehensive nurse-led follow-ups enabled the identification of key real-world challenges in oncology patients outside of clinical trials [14]. These included clinical deterioration, hospital admissions, treatment delays, and disease progression, often requiring additional diagnostic interventions. Seventy percent of patients had oligometastatic disease, and 43.3% received palliative care, factors that contributed to hospitalization and treatment cycle delays, ultimately impacting patient outcomes.

Despite these challenges, survey completion rates remained high, with 86.6% compliance at one month, 80% at three months, and 56.6% at six months. The median follow-up was 4.8 months. These findings are consistent with clinical trial data in which patients received CPI monotherapy or CPI combined with chemotherapy (ChT) [3,6,15,16,17,18].

### 4.1. Impact of Chemotherapy on HRQL

Patients with prior ChT exposure had poorer baseline health status, with a global health status score of 56, comparable to findings from previous studies [16]. In contrast, patients without prior ChT had a higher baseline score of 68, aligning with previous research suggesting that patients transitioning from ChT to immunotherapy may experience HRQL improvements over time [6]. In our study, patients previously treated with ChT demonstrated improved HRQL scores, reaching 63 at three months and 66 at six months.

These results would have been unthinkable a few years ago when most patients receiving immunotherapy had disseminated disease [3,19,20], typically experiencing significant deterioration and poor quality of life [13]. However, our findings suggest that patients receiving CPI-based therapies, either alone or in combination with ChT, experience significant QoL improvements at six months [6,17]. Notably, QoL outcomes did not differ significantly between CPI monotherapy and combination therapy groups, reinforcing the idea that adding ChT does not necessarily compromise QoL [15,16,21]. Symptom Burden and Toxicities

Fatigue was the most reported symptom, a finding consistent with previous studies [3,22,23]. Interestingly, patients receiving CPI monotherapy initially reported higher fatigue scores than combination therapy patients. However, by six months, fatigue scores declined significantly in CPI monotherapy patients. This suggests that CPI-related fatigue may be transient, possibly diminishing over time as patients adapt physiologically or experience long-term immunotherapy benefits.

Other CPI-associated toxicities included headache [24,25], skin reactions [26], diarrhea, nausea, and emesis [3,4,7,27]. However, our study reported nausea and emesis at low levels. Constipation scores were higher than diarrhea scores, and both constipation and pain scores remained stable throughout the six-month follow-up.

Previous research has suggested that immune-related adverse effects (irAEs) typically emerge 6–12 weeks after treatment initiation and subsequently decline [28]. However, our findings diverged from this trend, as constipation and pain scores remained consistent throughout the study, whereas fatigue improved over time, particularly in CPI monotherapy patients.

### 4.2. Study Limitations and Strengths

This study has several limitations, including a small sample size, tumor heterogeneity, and variability in immunotherapy regimens, all of which may have influenced the results. However, the primary strength of this study is its real-world applicability, providing valuable insights into HRQL outcomes beyond the structured setting of clinical trials.

## 5. Conclusions

This study demonstrates a positive impact on the quality of life for cancer patients treated with checkpoint inhibitors, as assessed using the QLQ-C30 Quality of Life Questionnaire. Checkpoint inhibitor therapy was linked to a significant delay in the onset of symptoms, with fatigue being the most commonly reported adverse effect.

The findings emphasize the importance and practicality of implementing health-related quality of life (HRQoL) questionnaires across diverse clinical settings. This study establishes a foundation for future research by pinpointing key challenges in gathering HRQoL data outside clinical trial contexts.

While more than 70% of participants reported no difficulty completing the questionnaires, nearly 27% faced challenges—mainly due to baseline limitations in reading or writing. Acknowledging these barriers is essential for improving data collection methods and enhancing patient compliance, thus supporting the routine integration of HRQoL assessments in oncology care.

## Figures and Tables

**Table 1:** QLQ-C30 Quality of Life questionnaire analyzed variables.

Variable Type	Variable	Description
Quantitative variable	Age	Over 18 years old.
	Presence or absence of symptoms	Degree of presence of symptoms.
	Assessment of quality of life in the week prior to treatment	Each patient specified the quality of life they had the week before treatment.
Qualitative variable	Sex	Male, Female.
	Oncological history	It was determined whether the patient had an oncological history other than the current tumor.
	History of autoimmune diseases	The presence of autoimmune diseases was recorded in each patient.
	Tumor histology	The histology of each patient’s tumor was specified.
	Tumor extension	Tumor extension was classified as localized, locally advanced, or metastatic disease.
	Treatment intention	The intention of the treatment that each patient would receive was defined as radical, neoadjuvant, adjuvant or palliative.
	Prior receipt of chemotherapy	It was recorded whether patients had previously received chemotherapy before being included in the study.
	Treatment that each patient will receive	It was specified whether patients received only IPC or a combination of chemotherapy and IPC.
	Drug	The drug that each patient would receive was recorded, including nivolumab, pembrolizumab, atezolizumab, durvalumab, avelumab or tremelimumab.
	Difficulty filling out the questionnaire	It was determined whether the patients had cognitive impairment, intellectual disability, or difficulty completing the questionnaire.
	Delay of treatment cycle	It was recorded whether patients experienced any delay in treatment cycles.
	Hospital admission	It was specified whether the patients required hospitalization due to their clinical status.

**Table 2:** Patients’ Characteristics.

Properties			Patients	
		Total	30	%
Demography characteristics	Male		17	56
	Female		13	43
Oncological history	Yes		8	26
	No		22	73
Autoimmune disease history	Yes		4	13
	No		26	86
Histology of tumor	Mesothelioma		2	6
	Adenocarcinoma		8	26
	Carcinoma		15	50
	Melanoma		4	13
	Leiomyyosarcoma		1	3
Spreading tumor	Located		5	17
	Locally advanced		4	13
	Metastatic		21	70
Treatment objective	Radical		8	26
	Neoadjuvant		4	13
	Adjuvant		5	16
	Paliative		13	43
Previous chemotherapy	Yes		12	40
	No		18	60
Current treatment	ChT-immunotherapy		12	40
	Immunotherapy		18	60
Signs and symptoms	Dyspnoea		13	43
	Pain		16	53
	Insomnia		16	53
	Asthenia		21	70
	Nausea		5	16
	Vomiting		3	10
	Diarrhea		9	30
	Constipation		13	43
	None		2	6
Quality of life assessment at the beginning of treatment (“0” very bad “7” very good)	0		0	0
	1		0	0
	2		2	6
	3		6	20
	4		6	20
	5		7	23
	6		5	16
	7		4	13
Difficulty filling out the questionnaire	Cognitive impairment		1	3
	Intellectual disability		1	3
	Difficulty reading/writing		6	20
	Without difficulty		22	73
Delay in treatment cycle at the start	yes		0	0
	no		30	100
Hospital admission at the beginning	yes		5	17
	no		25	83

**Table 3:** Symptom and functional scale score. Comparison of results based on whether they have received chemotherapy previously or not before entering the study.

	Results Receiving or Not Chemotherapy Previously												*p*-Value		
	Have Previously Received Chemotherapy			Have Not Received Chemotherapy			Have Previously Received Chemotherapy			Have Not Received Chemotherapy					
		Mean ± DE			Mean ± DE			Median [RIC]			Median [RIC]				
	Start	3 Months	6 Months	Start	3 Months	6 Months	Start	3 Months	6 Months	Start	3 Months	6 Months	Start	3 Months	6 Months
Fatigue	45.8 [22.5]	26.3 [16.7]	26.6 [16.8]	29.8 [22.5]	41.6 [30.7]	34.1 [30.2]	50 [38.8]	27.7 [55.5]	33.3 [27.7]	33.3 [38.8]	33.3 [52.7]	33.3 [38.8]	0.12	0.23	0.96
Nausea and vomiting	10.4 [15.2]	4.1 [11.7]	13.3 [29.8]	1 [4.1]	12.5 [21.5]	7.6 [12.9]	0 [29.1]	0 [0]	0 [33.3]	0 [0]	0 [83.3]	0 [16.6]	0.04	0.18	0.85
Pain	27 [26.6]	18.7 [18.7]	16.6 [20.4]	21.8 [19.9]	32.2 [25.4]	26.9 [25.9]	25 [50]	16.6 [66.6]	16.6 [33.3]	25 [33.3]	33.3 [45.8]	33.3 [33.3]	0.63	0.2	0.44
Dyspnoea	33.3 [25.1]	29.1 [33]	6.6 [14.9]	12.5 [20.6]	22.9 [23.4]	5.3 [22]	33.3 [50]	16.6 [66.6]	0 [16.6]	0 [33.3]	33.3 [33.3]	0 [33.3]	0.04	0.71	0.43
Insomnia	20.8 [24.8]	12.5 [24.8]	6.6 [14.9]	27 [27.8]	27 [25]	20.5 [21.6]	16.6 [33.3]	0 [25]	0 [16.6]	33.3 [33.3]	33.3 [33.3]	33.3 [33.3]	0.62	0.14	0.19
Loss of appetite	25 [23.5]	12.5 [24.8]	6.6 [14.9]	16.6 [29.8]	27 [34.8]	15.3 [32.2]	33.3 [33.3]	16.6 [33.3]	0 [16.6]	0 [33.3]	0 [25]	0 [16.6]	0.23	0.26	0.78
Constipation	25 [34.5]	8.3 [14.4]	33.3 [40.8]	22.9 [33.8]	20.8 [23.9]	20.5 [28.9]	66.6 [0]	0 [25]	33.3 [66.67]	0 [58.3]	16.6 [33.3]	0 [50]	0.91	0.2	0.47
Diarrhea	16.6 [25.1]	8.3 [15.4]	13.3 [18.2]	14.5 [27.1]	12.5 [20.6]	10.2 [16]	0 [33.3]	0 [25]	0 [33.3]	0 [33.3]	0 [33.3]	0 [33.3]	0.74	0.7	0.71
Financial difficulties	4.1 [11.7]	8.3 [23.5]	6.6 [14.9]	8.3 [14.9]	14.5 [27.1]	17.9 [35]	0 [0]	0 [0]	0 [16.6]	0 [25]	0 [25]	0 [33.3]	0.48	0.51	0.73
Phisical functioning	67.5 [24.2]	80.8 [16.4]	68 [25.5]	74.1 [27]	65.8 [29.6]	68.7 [32.4]	70 [25]	80 [18.3]	73.3 [46.6]	80 [43.3]	60 [50]	73.3 [60]	0.35	0.38	0.88
Role functioning	70.8 [31.8]	81.2 [18.7]	83.3 [28.8]	68.7 [36.9]	61.4 [35.3]	58.9 [38.8]	75 [62.5]	83.3 [33.3]	100 [41.6]	83.3 [62.5]	66.6 [66.6]	66.6 [83.3]	1	0.22	0.23
Emotional functioning	77 [18.2]	88.5 [23.1]	81.6 [17]	69.7 [22.1]	75 [19]	80.7 [19]	70.8 [31.2]	100 [14.5]	75 [33.3]	75 [37.5]	75 [25]	91.6 [33.3]	0.51	0.04	0.79
Cognitive functioning	81.2 [13.9]	85.4 [20.7]	90 [9.1]	85.4 [20]	73.9 [26.5]	65.3 [32.2]	83.3 [29.1]	100 [33.3]	83.3 [16.6]	100 [33.3]	75 [33.3]	66.6 [50]	0.35	0.31	0.1
Social functioning	70.8 [27.8]	87.5 [14.7]	83.3 [11.7]	78.1 [24.1]	72.9 [29.1]	67.9 [31.5]	75 [41.6]	91.6 [29.1]	83.3 [16.6]	83.3 [33.3]	83.3 [50]	66.6 [66.6]	0.5	0.25	0.51

**Table 4:** Comparison of outcomes when receiving checkpoint inhibitors alone or chemotherapy in combination with checkpoint inhibitors.

Comparison of Outcomes When Receiving Checkpoint Inhibitors Alone or Chemotherapy in Combination with Checkpoint Inhibitors													*p*-Value		
	Number of Patients has Received Checkpoint Inhibitors			Number of Patients Has Received Checkpoint Inhibitors in Combination with Chemotherapy			Number of Patients Has Received Checkpoint Inhibitors			Number of Patients Has Received Checkpoint Inhibitors in Combination with Chemotherapy					
		Mean ± DE			Mean ± DE			Median [RIC]			Median [RIC]				
	Start	3 Months	6 Months	Start	3 Months	6 Months	Start	3 Months	6 Months	Start	3 Months	6 Months	Start	3 Months	6 Months
Fatigue	41.4 [26]	33.3 [29.6]	30.5 [31.4]	24.6 [24]	41.9 [24]	35.1 [16.3]	44.4 [44.4]	33.3 [55.5]	27.7 [38.8]	22.2 [27.7]	33.3 [33.3]	33.3 [19.4]	0.11	0.36	0, 47
Náusea and vomiting	4.4 [9.8]	10 [22.5]	6.9 [13.2]	3.7 [11.1]	9.2 [12.1]	13.8 [26.7]	0 [0]	0 [16.6]	0 [12.5]	0 [0]	0 [16.6]	0 [29.1]	0.64	0.49	0.67
Pain	21.1 [20.3]	22.2 [23.2]	22.2 [25.9]	27.7 [25]	37 [23.2]	27.7 [22.7]	16.6 [33.3]	16.6 [50]	16.6 [33.3]	33.3 [50]	33.3 [41.6]	25 [29.1]	0.51	0.13	0.52
Dyspnoea	20 [21]	26.6 [28.7]	11.1 [21.7]	18.5 [29.3]	22.2 [23.5]	16.6 [18.2]	33.3 [33.3]	33.3 [66.6]	0 [25]	0 [50]	33.3 [33.3]	16.6 [33.3]	0.64	0.77	0.39
Insomnia	26.6 [25.8]	22.2 [24.1]	16.6 [17.40]	22.2 [28.8]	22.2 [24.1]	16.6 [27.8]	33.3 [33.3]	33.3 [33.3]	16.6 [33.3]	0 [50]	33.3 [33.3]	0 [41.6]	0.62	0.89	0.74
Loss of appetite	24.4 [29.4]	26.6 [38.2]	16.6 [33.3]	11.1 [23.5]	14.8 [17.5]	5.5 [13.6]	33.3 [33.3]	0 [66.6]	0 [25]	0 [16.6]	0 [33.3]	0 [8.3]	0.18	0.73	0.6
Constipation	24.4 [34.4]	13.3 [21]	25 [35.1]	22.2 [33.3]	22.2 [23.5]	22.2 [27.2]	0 [0]	0 [33.3]	0 [58.3]	0 [66.6]	33.3 [33.3]	16.6 [41.6]	0.83	0.30	0.95
Diarrhea	17.7 [30]	13.3 [21]	13.8 [17.1]	11.1 [16.6]	7.4 [14.6]	5.5 [13.6]	0 [0]	0 [33.3]	0 [33.3]	0 [33.3]	0 [16.6]	0 [8.3]	0.82	0.52	0.3
Financial difficulties	6.6 [13.8]	6.6 [18.6]	0 [0]	7.4 [14.6]	22.2 [33.3]	44.4 [40.3]	0 [16.6]	0 [66.6]	0 [0]	0 [16.6]	0 [66.6]	50 [75]	0.89	0.2	0.002
Phisical Functioning	69.3 [25.2]	74.6 [29.5]	71.1 [34.6]	76.2 [27.7]	64.4 [20.8]	63.3 [19.2]	73.3 [33.3]	80 [46.6]	83.3 [61.6]	80 [36.6]	53.3 [33.3]	70 [36.6]	0.4	0.19	0.36
Role Functioning	66.6 [36.7]	77.7 [30.6]	75 [37.2]	74 [32.3]	51.8 [28.1]	47.2 [32.3]	66.6 [50]	100 [33.3]	100 [58.3]	100 [66.6]	33.3 [41.6]	41.6 [58.3]	0.74	0.03	0.12
Emotional Functioning	76.1 [18.5]	80 [23.5]	82.6 [18.9]	65.7 [23.7]	78.7 [17.2]	77.7 [17.2]	75 [25]	91.6 [25]	91.6 [33.3]	58.3 [45.8]	66.6 [33.3]	66.6 [33.3]	0.21	0.6	0.55
Cognitive Functioning	84.4 [19.3]	83.3 [25.1]	79.1 [24.7]	83.3 [16.6]	68.5 [22.7]	58.3 [36.1]	83.3 [33.3]	100 [33.3]	83.3 [29.1]	83.3 [33.3]	66.6 [16.6]	66.6 [62.5]	0.72	0.07	0.16
Social Functioning	73.3 [23.4]	85.5 [26.6]	79.1 [28.5]	79.6 [28.5]	64.8 [19.4]	58.3 [22.9]	83.3 [50]	100 [16.6]	91.6 [33.3]	1001 [33.3]	66.6 [33.3]	58.3 [50]	0.38	0.007	0.08

**Table 5:** Global health status. Comparison of outcomes by age, sex, whether or not they have previously received ChT, and whether they have received CPI only or a combination of ChT and CPI.

Global Health Status Score								*p*-Value		
			Mean ± DE			Median [RIC]			*p* Value	
		Start	3 Months	6 Months	Start	3 Months	6 Months	Start	3 Months	6 Months
Age	65 years or younger	68 [22.1]	63.1 [29.6]	77.7 [27.6]	66.6 [39.5]	58.3 [60.4]	83.3 [50]	0.39	0.95	0.17
	Over 65 years old	60.4 [23.8]	64.5 [24.6]	61.1 [28.5]	62.5 [50]	66.6 [45.8]	58.3 [28.5]			
Sex	Male	51.2 [16.2]	61.1 [25.9]	68.5 [31.3]	50 [33.3]	66.6 [54.1]	83.3 [37.5]	0.002	1	0.96
	Female	79.5 [20.1]	68.6 [28.6]	70.3 [27.3]	83.3 [33.3]	66.6 [50]	66.6 [58.3]			
Previously received treatment	Have previously received chemotherapy	56.2 [25]	63.5 [23.5]	66.6 [19.5]	50 [45.8]	66.6 [43.7]	58.3 [29.1]	0.21	0.90	0.58
	Have not received chemotherapy previously	68.2 [21.3]	64 [28.8]	70.5 [32]	66.6 [31.2]	58.3 [60.4]	83.3 [58.3]			
Treatment	Checkpoint inhibitors	60 [22.3]	65 [30.8]	70.8 [30]	58.3 [50]	66.6 [66.6]	75 [47.9]	0.22	0.80	0.66
	Checkpoint inhibitors in combination with chemotherapy	71.2 [23.2]	62 [19.1]	66.6 [27.8]	66.6 [37.5]	66.6 [37.5]	75 [54.1]			

## Data Availability

The data supporting the results and findings of this study are available from the corresponding author. These data, due to confidentiality and ethical considerations, are not publicly available.
